# Personnel's Health Surveillance at Work: Effect of Age, Body Mass Index, and Shift Work on Mental Workload and Work Ability Index

**DOI:** 10.1155/2013/289498

**Published:** 2013-07-16

**Authors:** Shahram Safari, Jafar Akbari, Meghdad Kazemi, Mohammad Amin Mououdi, Behzad Mahaki

**Affiliations:** ^1^Department of Occupational Health Engineering, School of Health, Isfahan University of Medical Sciences, Isfahan 8174-73461, Iran; ^2^Department of Occupational Health Engineering, School of Health, Mazandaran University of Medical Sciences, Sari 48178-44718, Iran; ^3^Department of Biostatistics, School of Health, Isfahan University of Medical Sciences, Isfahan 8174-73461, Iran

## Abstract

*Introduction.* Two great changes in developed countries are taking place: populations are ageing and becoming increasingly overweight. Combination of these factors with shift work is a risk factor for work ability and mental workload that are dynamic processes which change greatly throughout an individual's work life. The aim of this study was to investigate mental workload and work ability in textile workers and to identify factors which affect work ability and mental workload. *Methods.* This cross-sectional study was carried out among 194 male workers in textile industry. Employees based on their job group and work conditions have been divided into 6 categories. They completed work ability index and mental workload questionnaires during three work shifts. Body mass index (BMI) and demographic details were recorded. *Results.* All of the participants rated their work ability as moderate with high mental workload. The mean WAI and mental workload in age group were significant. The mean BMI was 25.5 kg/m^2^ (standard deviation 4.1) and the mean age was 40.22 years. There was a statistically significant correlation between work ability index and shift work. *Conclusions.* Unlike the previous study, a decrease point in WAI started in early age that may be due to life-style work and another psychological factor; on the other hand, NASA-TLX revealed high score in six subscales that can be another reason for low WAI.

## 1. Introduction

Two great changes in developed countries are taking place: populations are ageing and becoming increasingly overweight [1]. The average weight of both men and women at the age of 45 years is 20% higher than it was 20 years age [2–4]; on the other hand, ageing workers can be found in almost all types of occupation [5]. The International Labor Organization (ILO) has estimated that by the year 2025, the proportion of individuals over the age of 55 years will be 32% in Europe, 30% in North America, 21% in Asia, and 17% in Latin America [6]. After 50 years of age, performance decrements become apparent. In UK, the Health and Safety Laboratory used the term “older workers” to refer to people 50 years of age [7, 8]. This is due to the following: with advancing age, decrements can be expected in aerobic capacity, general health, grip strength, lifting strength, balance, eyesight, hearing, reaction time, limb motility, and tolerance for paced work [8–10]. The work ability concept is a dynamic process that changes greatly for several reasons throughout an individual's work life. One of the main factors inducing change is aging and its effect on human resources [6]. Work ability is defined as the ability of a worker to perform his/her job, taking into account work demand and physical and mental conditions [11]. Work ability is a tool to identify workers at risk for imbalance between health, capabilities, and demands at work [12]. Improving work ability is one of the most effective ways to enhance the ability and to prevent disability and early retirement [13, 14]. Another factor that influenced work ability and consequence in early retirement is body mass index (BMI) in study by Lund et al., and Van den berg et al. underweight as well as Obesity compared to normal weight decreases the WAI score [12, 15]. Night shift work, as compared with day work, is a high risk for poor work ability, when combined with aging (44.5 years old) is significantly higher than the risk associated with their additive effect [16]. In a study by Capanni et al. in relation to working hours, WAI proved to be worse in shift workers than in day workers, and, particularly, in continuous 3-shift workers [17]. Measures of subjective workload show that what may appear to be a simple assignment is in fact quite demanding; on the other hand, jobs without specific demands will not require additional ergonomics attention or actions to keep older workers fit in the job [18–20]. These specific demands can be classified into (or combinations of) physical, mental, or psychosocial job characteristics [20]. Mental work load is the amount of effort that the mind can do in duty and requires input from such cognitive domains as concentration, memory, decision making, or attention [20, 21]. It refers to the relationship between resource supply and task demand [22]. There are three principal methods for measuring workload: physiological (heart rate and blood pressure as responses to stress such as that induced by physical activities), procedural (measuring time spent on secondary tasks), and perceptual or subjective (workload measurement that uses rating scales to evaluate participants' perceived workloads). Although physiological and procedural measurements may appear to be more accurate and objective, subjective measurement of workload has been reported to be less invasive, easier, and less expensive to obtain more easily reproduced, and of higher face validity [23]. A study by Bridger and Bennett among seafarers indicate that mental workload had a moderately high level of satisfaction with work performance, and all other scores were generally clustered around the midpoints of the rating scales [1].

Hence, little is known about the physical and mental demands of textile workers. The aim of the current investigation was to quantify the mental demands placed on textile workers during their normal daily work and shift activities in relation to their self-assessed work ability and mental workload. A second aim was to identify factors that affected mental workload and work ability index in textile workers.

## 2. Methods

This cross-sectional study was carried out in a textile industry in Isfahan, between September and November 2012. The participation in the study was voluntary. Approval was obtained from the company's ethics committee. Employees were selected from each job based on their job groups and work conditions, and they have been divided into 6 categories (Table 1).

Finally, one hundred and ninety-four employees completed questionnaires; then employees were invited for anthropometric measurements.

At the conclusion of the shift, participants returned to a briefing room where they completed two questionnaires.

### 2.1. Work Ability Index

Perceived work ability was measured by a questionnaire-based index composed of the following seven items: (i) current work ability compared with lifetime best (0–10 point); (ii) work ability in relation to both physical and mental demands of work (2–10 point); (iii) number of current diseases (1–7 points); (iv) estimated work impairment due to diseases (1–6 points); (v) sick leave during the past year (12 months) (1–5 points); (iv) own prognosis of work ability 2 years from now (1, 4, or 7 points); (vii) mental resources (enjoying daily task, activity and life spirit, and optimistic about the future) (1–4 points) [24]. Translating the WAI questionnaire into Persian and identifying its reliability and validity in Iran have been done by Abdolalizadeh et al. [25].

The cumulative index of WAI ranges from 7 to 49 points. It is divided into the following categories: poor (7–27 points), moderate (28–36 points), good (37–43 points), and excellent work ability (44–49 points) [26]. Subjects at or below 36 points were classified as having low work ability. Subjects at or above 37 points were classified as having satisfying work ability [26].

### 2.2. Mental Workload Questionnaire

Perceived mental workload was measured by NASA-TLX (the National Aeronautics and Space Administration Task Load Index) questionnaire [27]. NASA-TLX has six subscales, mental demand (MD), physical demand (PD), temporal demand (TD), performance (PF), effort (EF), and frustration (FR), which can be divided into three groups: characteristics of the task: mental, physical, and time demands; behavioral characteristics: performance and effort; individual characteristic: frustration. Each of the bipolar subscales of NASA-TLX consists of 20 five-point steps from 0–100 [27]. Each subject makes a paired comparison, deciding with all 15 possible pair combinations of the 6 dimensions which pair element is more important with regard to workload in the rated task. From the results, a rank order of the dimensions from 0–5 is derived by which the individual subscale scores of the rated task are weighted. By summing up the weighted subscale scores and dividing them by the sum of the weights (=15), the mean weighted workload score is obtained that indicates workload in percent [28].

### 2.3. Work Schedule

Participants were asked to report their shift schedules and these were categorized into, the following: morning shift from (7 am–15 pm), evening shift from (15 pm–23 pm), and night shift from (23 pm–7 am).

Anthropometric measurements were made at the end of the shift, using the following equipment and procedures according to standardized guidelines.

- Stature: volunteers removed their shoes before standing on a stadiometer (Invicta, Leicester, UK) with the feet together. Heels, buttocks, and scapulae were in contact with the stadiometer, and participants were instructed to look straight ahead to inhale steadily, and the measurement was taken to the nearest 0.1 cm.
- Mass: volunteers were barefoot, in general working clothes, with all items removed from their pockets. Weight was measured to the nearest 0.1 kg (Seca, Hamburg, Germany).

Body mass index was calculated using the formula weight/height^2^ and divided into four BMI categories: underweight was BMI ≤ 19 kg/m^2^, ideal weight was 19 < BMI ≤ 25 kg/m^2^, overweight was 25 < BMI ≤ 30, and severe overweight was BMI > 30 kg/m^2^ [29].

Finally, the information was analyzed using SPSS 20 and statistic tests, namely; multiple multivariate regression test has been used for relationships between individual factors such as age and BMI with WAI and NASA-TLX. Differences between three work shifts with mean WAI and NASA-TLX were analyzed by ANOVA test. Differences in WAI score between workplace groups were analyzed by ANOVA test. A *P* value less than 0.05 was considered to be statistically significant.

## 3. Results

Response was obtained from 194 subjects. The workers were between 24 and 62 years old, and their mean age was 40.22 ± 6.99 years. Differences in age between workplace groups were significant (one-way analysis of ANOVA, *P* < 0.001). All subjects who responded were men. The average period during which participants had been working at their current workplace was 15.17 years with range of 1–27 years. Smoking was divided into two levels: current smokers and nonsmokers. 7.2% of them were current smokers and 92.8% nonsmokers.  Table 2 summarizes participant anthropometric characteristics; mean body mass index (BMI) was 25.5 kg/m^2^ (overweight).

**Figure 1:**
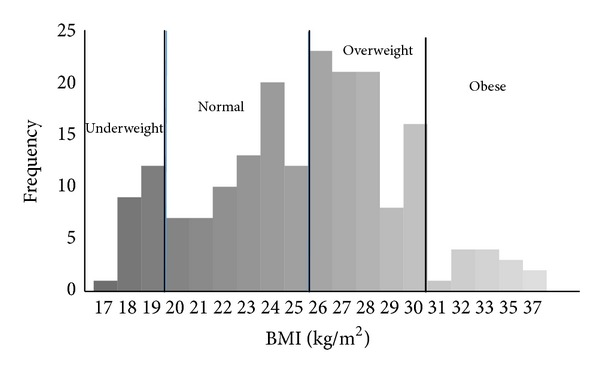
Distribution of BMI.

The percentage of the participants with underweight (BMI ≤ 19 kg/m^2^) was 11.3%, at a healthy weight (19 < BMI ≤ 25 kg/m^2^) was 35.6%, overweight (25 < BMI ≤ 30 kg/m^2^) was 45.9%, and severe overweight (BMI > 30 kg/m^2^) was 7.2% (Figure 1).

Multiple multivariate regression test showed that relationships between age and WAI were statistically significant (*P* = 0.007), but these relationships were not statistically significant with mental workload (*P* > 0.05).

Relationships between BMI with WAI and mental workload were not statistically significant (multiple multivariate regression test, *P* > 0.05).

ANOVA test revealed that relationships between work shifts and WAI were statistically significant (*P* < 0.05), and these relationships were not significant with mental workload (*P* > 0.05).

The average WAI score for all workers showed unsatisfying work ability. The lowest work ability was recorded among the doubling workers, followed by spinning workers, while the highest work ability was recorded among the supervisor workers. ANOVA test showed that after adjusting for age, there was a significant difference in the WAI scores between the workplace groups (*P* < 0.001) (Table 3).

The NASA-TLX mean ratings of the demands fell towards the high point except for time pressure and frustration that were placed at the midpoint of the scales, indicative of high demands as a percentage of maximum: mental demands 66.4%, SD 25.2%; physical demands 71.3%, SD 28%; time pressure 58.9%, SD 25.8%; effort 75.5%, SD 24.2%; frustration 44.5%, SD 30%; satisfaction with daily performance 73.5%, SD 23%.

The average NASA-TLX scores for job group indicative high mental workload. The highest mental workload was recorded among supervisors and the lowest was recorded among office workers (Table 3).

The result of one-way analysis of ANOVA shown between WAI and NASA-TLX in job groups was statistically significant (*P* = 0.01).

## 4. Discussion

**Figure 2:**
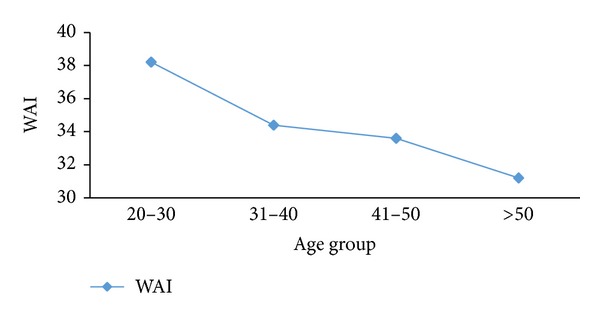
Mean WAI among age groups.

The perceived work ability of textile workers, as assessed by the WAI, decreased at an early age. The first significant turning point was observed after 30 years of age, as shown in Figure 2; at age 20–30 years mean WAI was 38.2 that placed in good work ability and after 30 years of age mean WAI was decreased that placed in poor work ability, And the second, steeper decrease occurred after 40 years of age, and this can because changes in physical work capacity that the maximal oxygen consumption (*V*O_2_max) shows linear decline with age. Generally, the changes in physical capacity in relation to aging are often difficult to distinguish because work and living habits can accelerate or slow down such changes. Regular physical exercise can keep physical capacity nearly unchanged between 45 and 65 years [6]. Changes in musculoskeletal capacity can also be pronounced after the age of 45–50 years. In a follow-up study, physically demanding jobs decreased by 40–50% during a 10-year period. The decrease was 4-5 times greater than in cross-sectional studies [30].

Multiple multivariate regression test showed that age relationships were not statistically significant with mental workload and this is maybe result in NASA-TLX scores are significantly affected by both mental and physical workload and among six subscale only one subscale related to physical demand and this subscale may affect by age but determining the total score related to six subscale. Until now, extensive research has been performed on the separate assessment of physical and mental workloads, but there are no validated techniques available for measuring overall workload in multitask situations that involve substantial levels of both physical and mental workloads. Finding of this research was similar to Bridger and colleague's study among seafarers [1].

Relationships between BMI with WAI and mental workload were not statistically significant.

As shown in Table 4, weight gain in adult men is normal up to the age of 50 years due to the presence of a small, positive energy balance. It tails off at about the age of 50 years. At the age of 45 years, the average body mass of men and women is 20% higher than it was 20 years before [31]. The best predictor of WAI score was the interaction between BMI and age, in the expected direction. Older personnel with high BMI reported lower work ability. 

Shift work implies that working hour differs from the traditional diurnal work period (7 pm and 6 am) [32, 33]. Shift work may cause several problems in physical and mental health and work ability in workers [34]. In relation to shift work, WAI proved to be worse in shift workers than in day workers. In morning shift, mean WAI score was 35.1 (±4.9); in evening shift, it was 32.8 (±6.2); in night shift, it was 33 (±4.9). Similar findings have been reported in the study by Costa et al. among health care workers; the result revealed that shift workers showed a more pronounced decrease of WAI over the years compared to their colleagues day workers [35].

On the other hand, NASA-TLX score among night shift workers had a bigger mark than day shift workers. Studies of shift work have demonstrated that working shifts may lead to health complaints and social problems [36]. Alternating the working hours has decreased the amount of time available for rest and recovery within the context of current working hours, and this factor can affect mental workload during shift work.

The average WAI score among textile industry workers less than 37 points indicated unsatisfying work ability. In the present study, the average WAI score for all workers showed unsatisfying work ability (WAI < 37). These results are lower than the Finnish reference data in mentally demanding work (mean 39) [37]. This may result in textile industry due to the nature of the work in which the physical conditions of the workplace consist of stressors such as harmful physical agents (noise, lighting), harmful chemical agents (air borne cotton dust, ventilation condition of saloons), ergonomic risks (lifting and handling equipment, bad posture during working), and another occupational hazard that can affect WAI. The result is similar to the study by Aittomäki et al. which was conducted among municipal employees [38]. And comparative with studies in an oil company in Croatia [26], among fire fighter in Belgium [39], and in constructer worker in Netherland [40] that WAI was satisfying for all workers.

The average NASA-TLX scores for job group indicated high mental workload. The lowest mental workload was recorded among office workers and the highest was recorded among the supervisor workers with mean 70.8 (±17.5) and 89.5 (±7.8), respectively, and consist with study by Jin et al. among drivers in Japan that result indicated high mental demand such as this study [41].

The result of one-way analysis of ANOVA showed that relationships between WAI and NASA-TLX in job group were statistically significant (*P* = 0.01). As shown in Table 3, mean WAI in all job groups was low, and mean NASA-TLX score was high; these two variables have direct inverse effect that was revealed in a study by Sjögren-Rönkä among office workers (2002), and the result showed that low mental demand at work was directly related to higher work ability [42].

## 5. Conclusion

The results of the current study showed that the most important factor that influenced work ability among textile workers was age. Unlike the previous study, a decrease point in WAI score started in early age that may be due to life-style work and another psychological factor; on the other hand, NASA-TLX revealed high score in six subscales that can be another reason for low WAI. The result indicated that NASA-TLX score was more affected by working conditions in contrast to individual factors such as age and BMI.

## Figures and Tables

**Table 1:** Distribution of the respondents according to their job groups.

Job	No.	%
Spinning	50	25.8
Weaving	55	28.4
Repair	30	15.5
Supervisor	10	5.2
Office	15	7.7
Doubling	34	17.0

**Table 2:** Anthropometric characteristics.

	Mean	SD	Range
Stature (cm)	170.3	7.4	143–190
Mass (kg)	73.87	12.7	47–105
BMI (kg/m^2^)	25.5	4.1	17–37

**Table 3:** Mean (±SD) WAI and mental workload score.

Job	WAI	SD	Mental workload	SD
Spinning	32.7	5.6	72.8	15.9
Weaving	35.9	4.8	74.3	18.3
Repair	34.4	7.1	82.7	9.6
Supervisor	36.3	3.2	89.5	7.8
Office	34	6	70.8	17.5
Doubling	31.9	5.8	78.7	13.4
Total score	34	5.8	76.5	15.8

**Table 4:** Mean (SD) BMI in different age groups.

Age groups	BMI	SD
20–30	24	4.1
31–40	25	4.3
41–50	26.5	3.8
>50	24.8	3.3
